# Supplementary material for: Association of Circadian Clock Gene Expression with Pediatric/Adolescent Asthma and Its Comorbidities
Source: Int J Mol Sci. 2023 Apr 19;24(8):7477. doi: 10.3390/ijms24087477 (PMC10138904; doi:10.3390/ijms24087477)
Supplement: Supplementary file 1 [file ijms-24-07477-s001.zip › ijms-2304314-supplementary.pdf]

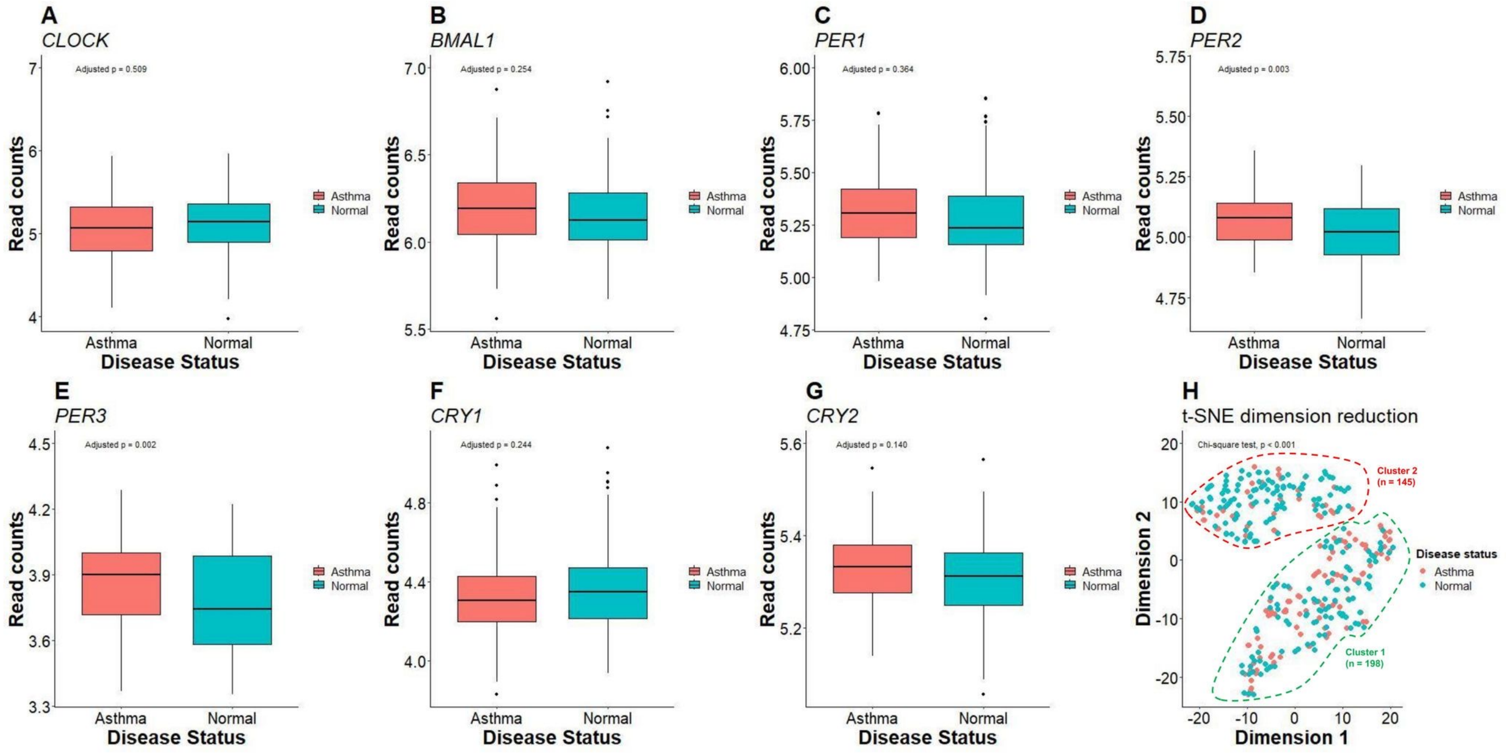

Supplementary Figure S1. (**A-G**) The boxplots comparing the read counts of each core clock gene in blood samples of patients with and without asthma. (**H**) t-SNE dimension reduction of expression matrix of 7 clock genes showed two distinct clusters with higher frequency of asthma patients in cluster 1 ($p<0.001$).

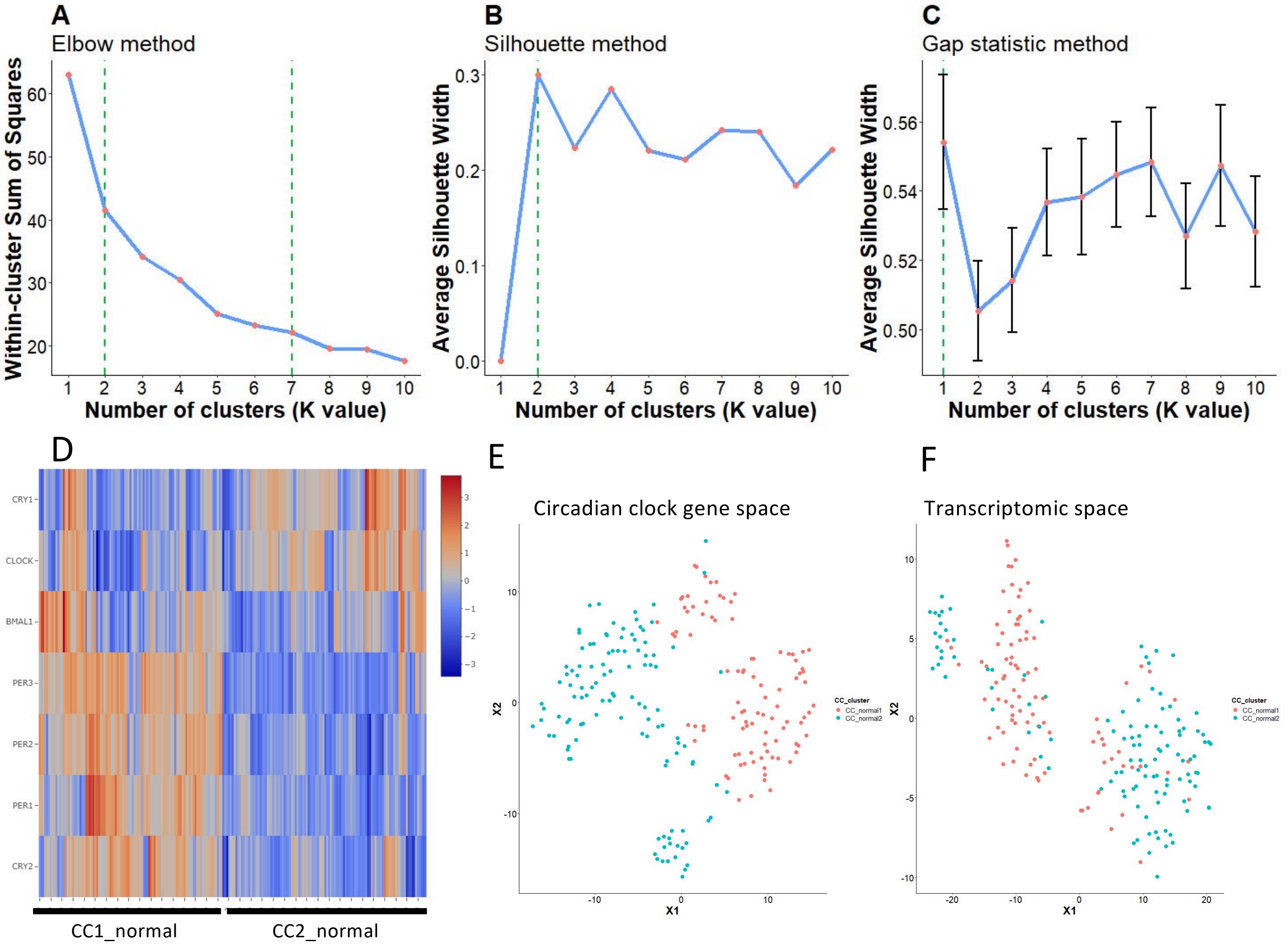

Supplementary Figure S2. (**A**) The Elbow method shows significant drops of within-cluster sum of squares from K=2 to K=7 while the silhouette and gap statistic methods suggest K=2 and K=1 is the optimal value, respectively (**B**, **C**). We chose K=2 as the optimal number of CC clusters in the control cohort. (**D**) The heatmap showing the expression pattern of the core clock genes. t-SNE dimension reduction illustrates that the 2 CC subtypes form a distinct cluster in both circadian clock space (**E**) and whole transcriptomic space (**F**).

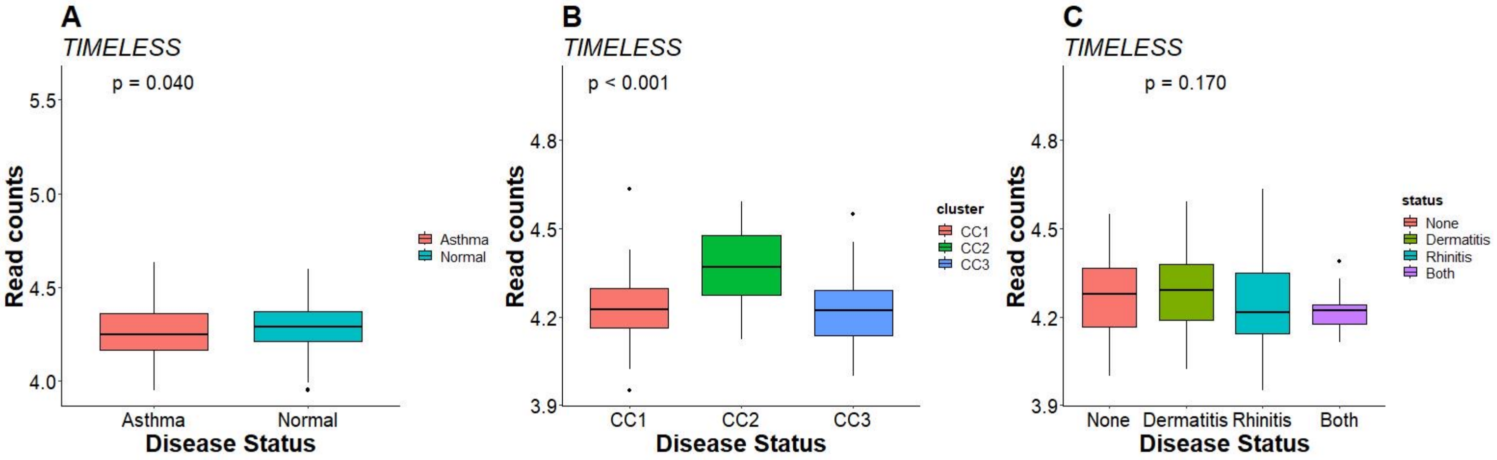

Supplementary Figure S3. The boxplots comparing the read counts of TIMELESS in blood samples of patients with and without asthma (A), of asthma patients in each CCs (B), and of each condition of comorbidity (C).

Supplementary Table S1. The results of ANOVA tests with Bonferroni multiple hypothesis correction to compare activities of each pathway in the 3 CC subtypes.

Pathway	Raw p-value	Adjusted p-value
KEGG_ABC_TRANSPORTERS	2.43E-09	4.51E-07
KEGG_ACUTE_MYELOID_LEUKEMIA	1.11E-10	2.06E-08
KEGG_ADHERENS_JUNCTION	2.05E-11	3.81E-09
KEGG_ADIPOCYTOKINE_SIGNALING_PATHWAY	2.25E-12	4.18E-10
KEGG_ALANINE_ASPARTATE_AND_GLUTAMATE_METABOLISM	0.36274736	1
KEGG_ALDOSTERONE_REGULATED_SODIUM_REABSORPTION	8.83E-19	1.64E-16
KEGG_ALLOGRAFT_REJECTION	8.54E-05	0.015886635
KEGG_ALPHA_LINOLENIC_ACID_METABOLISM	2.83E-07	5.26E-05
KEGG_ALZHEIMERS_DISEASE	3.40E-12	6.32E-10
KEGG_AMINO_SUGAR_AND_NUCLEOTIDE_SUGAR_METABOLISM	4.04E-13	7.52E-11
KEGG_AMINOACYL_TRNA_BIOSYNTHESIS	1.80E-17	3.35E-15
KEGG_AMYOTROPHIC_LATERAL_SCLEROSIS_ALS	3.02E-12	5.62E-10
KEGG_ANTIGEN_PROCESSING_AND_PRESENTATION	5.96E-06	0.00110772
KEGG_APOPTOSIS	3.59E-09	6.68E-07
KEGG_ARACHIDONIC_ACID_METABOLISM	5.58E-13	1.04E-10
KEGG_ARGININE_AND_PROLINE_METABOLISM	0.046946374	1
KEGG_ARRHYTHMOGENIC_RIGHT_VENTRICULAR_CARDIOMYOPATHY_ARVC	1.49E-21	2.76E-19
KEGG_ASCORBATE_AND_ALDARATE_METABOLISM	0.046632359	1
KEGG_ASTHMA	1.93E-06	3.59E-04
KEGG_AUTOIMMUNE_THYROID_DISEASE	0.002351618	0.43740103
KEGG_AXON_GUIDANCE	1.03E-23	1.92E-21
KEGG_B_CELL_RECEPTOR_SIGNALING_PATHWAY	1.11E-07	2.06E-05
KEGG_BASAL_CELL_CARCINOMA	3.86E-12	7.17E-10
KEGG_BASAL_TRANSCRIPTION_FACTORS	3.27E-20	6.08E-18
KEGG_BASE_EXCISION_REPAIR	5.43E-07	1.01E-04
KEGG_BETA_ALANINE_METABOLISM	5.80E-12	1.08E-09
KEGG_BIOSYNTHESIS_OF_UNSATURATED_FATTY_ACIDS	0.001632581	0.303660115
KEGG_BLADDER_CANCER	4.14E-19	7.70E-17
KEGG_BUTANOATE_METABOLISM	3.69E-04	0.068673805
KEGG_CALCIUM_SIGNALING_PATHWAY	3.21E-19	5.98E-17
KEGG_CARDIAC_MUSCLE_CONTRACTION	2.65E-04	0.049349975
KEGG_CELL_ADHESION_MOLECULES_CAMS	2.58E-15	4.80E-13
KEGG_CELL_CYCLE	1.43E-16	2.66E-14
KEGG_CHEMOKINE_SIGNALING_PATHWAY	2.41E-12	4.48E-10
KEGG_CHRONIC_MYELOID_LEUKEMIA	3.43E-10	6.38E-08
KEGG_CIRCADIAN_RHYTHM_MAMMAL	1.71E-12	3.17E-10
KEGG_CITRATE_CYCLE_TCA_CYCLE	4.40E-10	8.19E-08
KEGG_COLORECTAL_CANCER	1.26E-07	2.35E-05
KEGG_COMPLEMENT_AND_COAGULATION_CASCADES	3.14E-13	5.84E-11
KEGG_CYSTEINE_AND_METHIONINE_METABOLISM	1.06E-11	1.97E-09
KEGG_CYTOKINE_CYTOKINE_RECEPTOR_INTERACTION	2.70E-15	5.01E-13
KEGG_CYTOSOLIC_DNA_SENSING_PATHWAY	1.75E-04	0.032573963
KEGG_DILATED_CARDIOMYOPATHY	6.13E-19	1.14E-16
KEGG_DNA_REPLICATION	1.48E-16	2.75E-14
KEGG_DORSO_VENTRAL_AXIS_FORMATION	2.66E-13	4.94E-11
KEGG_DRUG_METABOLISM_CYTOCHROME_P450	5.06E-10	9.41E-08
KEGG_DRUG_METABOLISM_OTHER_ENZYMES	1.00E-05	0.001867933
KEGG_ECM_RECEPTOR_INTERACTION	2.47E-14	4.60E-12
KEGG_ENDOCYTOSIS	6.20E-14	1.15E-11
KEGG_ENDOMETRIAL_CANCER	4.16E-11	7.74E-09
KEGG_EPITHELIAL_CELL_SIGNALING_IN_HELICOBACTER_PYLORI_INFECTION	6.12E-10	1.14E-07
KEGG_ERBB_SIGNALING_PATHWAY	2.65E-13	4.93E-11
KEGG_ETHER_LIPID_METABOLISM	0.039671837	1
KEGG_FATTY_ACID_METABOLISM	1.08E-10	2.01E-08
KEGG_FC_EPSILON_RI_SIGNALING_PATHWAY	8.18E-09	1.52E-06
KEGG_FC_GAMMA_R_MEDIATED_PHAGOCYTOSIS	6.35E-09	1.18E-06
KEGG_FOCAL_ADHESION	3.74E-23	6.95E-21
KEGG_FOLATE_BIOSYNTHESIS	0.003264078	0.607118469
KEGG_FRUCTOSE_AND_MANNOSE_METABOLISM	0.00246817	0.459079532
KEGG_GALACTOSE_METABOLISM	1.13E-10	2.11E-08
KEGG_GAP_JUNCTION	1.05E-24	1.96E-22

KEGG_GLIOMA	9.09E-14	1.69E-11
KEGG_GLUTATHIONE_METABOLISM	1.45E-04	0.02700266
KEGG_GLYCEROLIPID_METABOLISM	7.48E-08	1.39E-05
KEGG_GLYCEROPHOSPHOLIPID_METABOLISM	3.16E-05	0.005871687
KEGG_GLYCINE_SERINE_AND_THREONINE_METABOLISM	7.09E-12	1.32E-09
KEGG_GLYCOLYSIS_GLUCONEOGENESIS	3.92E-10	7.28E-08
KEGG_GLYCOSAMINOGLYCAN_BIOSYNTHESIS_CHONDROITIN_SULFATE	8.66E-09	1.61E-06
KEGG_GLYCOSAMINOGLYCAN_BIOSYNTHESIS_HEPARAN_SULFATE	2.43E-10	4.53E-08
KEGG_GLYCOSAMINOGLYCAN_BIOSYNTHESIS_KERATAN_SULFATE	0.968074447	1
KEGG_GLYCOSAMINOGLYCAN_DEGRADATION	1.12E-10	2.09E-08
KEGG_GLYCOSPHINGOLIPID_BIOSYNTHESIS_GANGLIO_SERIES	0.030469141	1
KEGG_GLYCOSPHINGOLIPID_BIOSYNTHESIS_GLOBO_SERIES	5.37E-10	9.98E-08
KEGG_GLYCOSPHINGOLIPID_BIOSYNTHESIS_LACTO_AND_NEOLACTO_SERIES	8.35E-04	0.155376724
KEGG_GLYCOSYLPHOSPHATIDYLINOSITOL_GPI_ANCHOR_BIOSYNTHESIS	1.61E-10	2.99E-08
KEGG_GLYOXYLATE_AND_DICARBOXYLATE_METABOLISM	8.89E-05	0.016527561
KEGG_GNRH_SIGNALING_PATHWAY	2.16E-12	4.02E-10
KEGG_GRAFT_VERSUS_HOST_DISEASE	0.024574377	1
KEGG_HEDGEHOG_SIGNALING_PATHWAY	3.97E-10	7.38E-08
KEGG_HEMATOPOIETIC_CELL_LINEAGE	0.010813071	1
KEGG_HISTIDINE_METABOLISM	1.31E-04	0.024288505
KEGG_HOMOLOGOUS_RECOMBINATION	2.14E-15	3.98E-13
KEGG_HUNTINGTONS_DISEASE	9.30E-13	1.73E-10
KEGG_HYPERTROPHIC_CARDIOMYOPATHY_HCM	2.19E-20	4.08E-18
KEGG_INOSITOL_PHOSPHATE_METABOLISM	1.01E-09	1.89E-07
KEGG_INSULIN_SIGNALING_PATHWAY	7.01E-12	1.30E-09
KEGG_INTESTINAL_IMMUNE_NETWORK_FOR_IGA_PRODUCTION	6.83E-09	1.27E-06
KEGG_JAK_STAT_SIGNALING_PATHWAY	7.49E-06	0.001393054
KEGG_LEISHMANIA_INFECTION	0.001170784	0.217765765
KEGG_LEUKOCYTE_TRANSENDOTHELIAL_MIGRATION	8.05E-11	1.50E-08
KEGG_LIMONENE_AND_PINENE_DEGRADATION	3.76E-11	6.99E-09
KEGG_LINOLEIC_ACID_METABOLISM	8.46E-11	1.57E-08
KEGG_LONG_TERM_DEPRESSION	7.52E-19	1.40E-16
KEGG_LONG_TERM_POTENTIATION	6.64E-14	1.24E-11
KEGG_LYSINE_DEGRADATION	5.46E-11	1.01E-08
KEGG_LYSOSOME	1.02E-06	1.90E-04
KEGG_MAPK_SIGNALING_PATHWAY	6.83E-12	1.27E-09
KEGG_MATURITY_ONSET_DIABETES_OF_THE_YOUNG	8.87E-14	1.65E-11
KEGG_MELANOGENESIS	7.07E-20	1.32E-17
KEGG_MELANOMA	5.72E-22	1.06E-19
KEGG_METABOLISM_OF_XENOBIOTICS_BY_CYTOCHROME_P450	8.51E-11	1.58E-08
KEGG_MISMATCH_REPAIR	1.78E-18	3.30E-16
KEGG_MTOR_SIGNALING_PATHWAY	2.34E-13	4.36E-11
KEGG_N_GLYCAN_BIOSYNTHESIS	4.96E-11	9.22E-09
KEGG_NATURAL_KILLER_CELL_MEDIATED_CYTOTOXICITY	7.87E-09	1.46E-06
KEGG_NEUROACTIVE_LIGAND_RECEPTOR_INTERACTION	4.11E-17	7.65E-15
KEGG_NEUROTROPHIN_SIGNALING_PATHWAY	1.56E-11	2.91E-09
KEGG_NICOTINATE_AND_NICOTINAMIDE_METABOLISM	2.83E-05	0.005263922
KEGG_NITROGEN_METABOLISM	3.76E-05	0.007001374
KEGG_NOD_LIKE_RECEPTOR_SIGNALING_PATHWAY	3.09E-07	5.75E-05
KEGG_NON_HOMOLOGOUS_END_JOINING	2.84E-17	5.29E-15
KEGG_NON_SMALL_CELL_LUNG_CANCER	1.20E-13	2.23E-11
KEGG_NOTCH_SIGNALING_PATHWAY	1.21E-12	2.24E-10
KEGG_NUCLEOTIDE_EXCISION_REPAIR	7.37E-21	1.37E-18
KEGG_O_GLYCAN_BIOSYNTHESIS	0.029780809	1
KEGG_OLFACTORY_TRANSDUCTION	3.11E-06	5.79E-04
KEGG_ONE_CARBON_POOL_BY_FOLATE	4.10E-13	7.63E-11
KEGG_OOCYTE_MEIOSIS	3.53E-12	6.56E-10
KEGG_OTHER_GLYCAN_DEGRADATION	1.05E-07	1.96E-05
KEGG_OXIDATIVE_PHOSPHORYLATION	4.48E-16	8.33E-14
KEGG_P53_SIGNALING_PATHWAY	1.59E-08	2.96E-06
KEGG_PANCREATIC_CANCER	5.56E-13	1.03E-10
KEGG_PANTOTHENATE_AND_COA_BIOSYNTHESIS	3.45E-07	6.41E-05
KEGG_PARKINSONS_DISEASE	2.43E-15	4.53E-13
KEGG_PATHOGENIC_ESCHERICHIA_COLI_INFECTION	3.14E-10	5.83E-08

KEGG_PATHWAYS_IN_CANCER	1.08E-19	2.01E-17
KEGG_PENTOSE_AND_GLUCURONATE_INTERCONVERSIONS	0.29717364	1
KEGG_PENTOSE_PHOSPHATE_PATHWAY	2.03E-10	3.77E-08
KEGG_PEROXISOME	2.65E-14	4.93E-12
KEGG_PHENYLALANINE_METABOLISM	2.13E-13	3.96E-11
KEGG_PHOSPHATIDYLINOSITOL_SIGNALING_SYSTEM	4.07E-11	7.58E-09
KEGG_PORPHYRIN_AND_CHLOROPHYLL_METABOLISM	0.085773932	1
KEGG_PPAR_SIGNALING_PATHWAY	7.53E-15	1.40E-12
KEGG_PRIMARY_BILE_ACID_BIOSYNTHESIS	2.44E-09	4.55E-07
KEGG_PRIMARY_IMMUNODEFICIENCY	9.29E-13	1.73E-10
KEGG_PRION_DISEASES	1.67E-12	3.10E-10
KEGG_PROGESTERONE_MEDIATED_OOCYTE_MATURATION	1.12E-13	2.09E-11
KEGG_PROPANOATE_METABOLISM	1.56E-16	2.90E-14
KEGG_PROSTATE_CANCER	1.59E-13	2.95E-11
KEGG_PROTEASOME	3.76E-18	7.00E-16
KEGG_PROTEIN_EXPORT	9.44E-21	1.76E-18
KEGG_PROXIMAL_TUBULE_BICARBONATE_RECLAMATION	0.006434653	1
KEGG_PURINE_METABOLISM	7.71E-09	1.43E-06
KEGG_PYRIMIDINE_METABOLISM	8.19E-15	1.52E-12
KEGG_PYRUVATE_METABOLISM	2.53E-09	4.70E-07
KEGG_REGULATION_OF_ACTIN_CYTOSKELETON	3.00E-16	5.57E-14
KEGG_REGULATION_OF_AUTOPHAGY	2.25E-05	0.004175859
KEGG_RENAL_CELL_CARCINOMA	2.35E-13	4.37E-11
KEGG_RENIN_ANGIOTENSIN_SYSTEM	1.57E-08	2.93E-06
KEGG_RETINOL_METABOLISM	1.91E-11	3.54E-09
KEGG_RIBOFLAVIN_METABOLISM	5.08E-11	9.46E-09
KEGG_RIBOSOME	4.99E-17	9.28E-15
KEGG_RIG_I_LIKE_RECEPTOR_SIGNALING_PATHWAY	3.80E-07	7.08E-05
KEGG_RNA_DEGRADATION	7.70E-18	1.43E-15
KEGG_RNA_POLYMERASE	6.23E-15	1.16E-12
KEGG_SELENOAMINO_ACID_METABOLISM	1.03E-10	1.92E-08
KEGG_SMALL_CELL_LUNG_CANCER	2.17E-07	4.04E-05
KEGG_SNARE_INTERACTIONS_IN_VESICULAR_TRANSPORT	2.52E-10	4.68E-08
KEGG_SPHINGOLIPID_METABOLISM	0.009312565	1
KEGG_SPLICEOSOME	2.78E-21	5.18E-19
KEGG_STARCH_AND_SUCROSE_METABOLISM	8.00E-06	0.001487139
KEGG_STEROID_BIOSYNTHESIS	6.70E-11	1.25E-08
KEGG_STEROID_HORMONE_BIOSYNTHESIS	4.59E-08	8.54E-06
KEGG_SULFUR_METABOLISM	8.12E-06	0.001510241
KEGG_SYSTEMIC_LUPUS_ERYTHEMATOSUS	0.001657303	0.308258365
KEGG_T_CELL_RECEPTOR_SIGNALING_PATHWAY	5.05E-11	9.40E-09
KEGG_TASTE_TRANSDUCTION	0.336674854	1
KEGG_TAURINE_AND_HYPOTAURINE_METABOLISM	1.44E-11	2.67E-09
KEGG_TERPENOID_BACKBONE_BIOSYNTHESIS	6.16E-16	1.15E-13
KEGG_TGF_BETA_SIGNALING_PATHWAY	2.78E-15	5.17E-13
KEGG_THYROID_CANCER	7.78E-05	0.014471596
KEGG_TIGHT_JUNCTION	2.03E-21	3.77E-19
KEGG_TOLL_LIKE_RECEPTOR_SIGNALING_PATHWAY	4.26E-08	7.92E-06
KEGG_TRYPTOPHAN_METABOLISM	7.65E-05	0.01422828
KEGG_TYPE_I_DIABETES_MELLITUS	0.244163992	1
KEGG_TYPE_II_DIABETES_MELLITUS	1.35E-15	2.51E-13
KEGG_TYROSINE_METABOLISM	9.30E-08	1.73E-05
KEGG_UBIQUITIN_MEDIATED_PROTEOLYSIS	3.35E-14	6.22E-12
KEGG_VALINE_LEUCINE_AND_ISOLEUCINE_BIOSYNTHESIS	4.71E-09	8.77E-07
KEGG_VALINE_LEUCINE_AND_ISOLEUCINE_DEGRADATION	3.42E-15	6.36E-13
KEGG_VASCULAR_SMOOTH_MUSCLE_CONTRACTION	2.95E-13	5.49E-11
KEGG_VASOPRESSIN_REGULATED_WATER_REABSORPTION	1.08E-11	2.00E-09
KEGG_VEGF_SIGNALING_PATHWAY	1.89E-12	3.52E-10
KEGG_VIBRIO_CHOLERAE_INFECTION	3.13E-09	5.82E-07
KEGG_VIRAL_MYOCARDITIS	0.230752193	1
KEGG_WNT_SIGNALING_PATHWAY	4.95E-13	9.20E-11
